# Supplementary material for: The Prevalence of Legal Performance-Enhancing Substance Use and Potential Cognitive and or Physical Doping in German Recreational Triathletes, Assessed via the Randomised Response Technique
Source: Sports (Basel). 2019 Nov 26;7(12):241. doi: 10.3390/sports7120241 (PMC6956052; doi:10.3390/sports7120241)
Supplement: Supplementary file 1 [file sports-07-00241-s001.pdf]

sports

MDPI

Supplementary Data

The Prevalence of Legal Performance-Enhancing Substance Use and Potential Cognitive and or Physical Doping in German Recreational Triathletes, Assessed via the 'Randomised Response Technique'

Sebastian Seifarth [1,2], Pavel Dietz [3], Alexander C. Disch [1], Martin Engelhardt [4] and Stefan Zwingenberger [1*]

[1] Department of Sports Medicine at the University Center for Orthopedics and Traumatology, University Medicine Carl Gustav Carus, Technical University Dresden, Dresden, Germany
[2] Bundeswehr hospital Ulm, Ulm, Germany
[3] Institute of Occupational, Social and Environmental Medicine, University of Mainz, Mainz, Germany
[4] Department of Orthopedics, Trauma and Hand Surgery, Klinikum Osnabrück, Osnabrück, Germany
* Correspondence: Stefan.Zwingenberger@uniklinikum-dresden.de; Tel.: +49-1727908318

Table S1. Influence of the longest distance raced on doping prevalence.

Variable (longest distance raced over last 12 month)			'yes'	'no'	a	$\hat{\pi}_s$ (%)	SE($\hat{\pi}_s$)	95% CI	Power
None	pd	n = 287	52	235	0.181	2.7	0.034	-3.9-9.3	0.22
	cd	n = 298	79	219	0.265	15.2	0.038	7.7-22.7	1
Super sprint or sprint	pd	n = 550	115	435	0.209	6.9	0.026	1.8-11.9	0.88
	cd	n = 567	136	431	0.24	11.4	0.027	6.2-16.7	1
Olympic distance	pd	n = 475	107	368	0.225	9.3	0.029	3.7-14.9	0.96
	cd	n = 494	113	381	0.229	9.8	0.028	4.3-15.3	0.98
Half-distance or long-distance	pd	n = 491	105	386	0.214	7.6	0.028	2.2-13.0	0.9
	cd	n = 509	95	414	0.187	3.5	0.026	-1.5-8.6	0.42

The given 'yes' and 'no' answers, for physical doping (pd, grey-background) and cognitive doping (cd, white-background) are presented separately for different race distances, as are the percentage of 'yes' answers from the sub-population. For all 'yes' answers (a), the standard error (SE), confidence interval (CI) and the calculated prevalence estimates ($\hat{\pi}_s$) are provided. A Post-hoc power analyses (Power) was performed to verify the results.

Table S2. Factors associated with physical and cognitive doping.

Variable			'yes'	'no'	a	$\hat{\pi}_s$ (%)	SE($\hat{\pi}_s$)	95% CI	Power
**Gender**									
female	pd	n = 419	84	335	0.201	5.6	0.029	-0.1-11.3	0.66
	cd	n = 437	110	327	0.252	13.2	0.031	7.1-19.3	1
male	pd	n = 1,381	295	1,086	0.214	7.5	0.016	4.3-10.8	1
	cd	n = 1,428	312	1,116	0.219	8.3	0.016	5.1-11.5	1
**A-level** (German diploma, that qualifies the holder for university admission)									

yes	pd	n = 1,264	269	995	0.213	7.4	0.017	4.1-10.8	1
	cd	n = 1,306	318	988	0.244	12.0	0.018	8.5-15.5	1
no	pd	n = 524	102	422	0.195	4.7	0.026	-0.4-9.8	0.61
	cd	n = 547	99	448	0.181	2.7	0.025	-2.1-7.5	0.32
**Years doing endurance sports#**									
≤ 10 years	pd	n = 963	198	765	0.256	6.3	0.019	2.5-10.1	0.96
	cd	n = 1,000	217	783	0.217	8.0	0.019	4.2-11.8	1
> 10 years	pd	n = 658	149	509	0.226	9.4	0.024	4.7-14.2	1
	cd	n = 679	158	521	0.233	10.4	0.024	5.6-15.1	1
**Training in a group**									
yes	pd	n = 907	181	726	0.2	5.4	0.02	1.6-9.3	0.89
	cd	n = 936	223	713	0.238	11.2	0.021	7.1-15.3	1
no	pd	n = 883	195	688	0.221	8.6	0.021	4.5-12.7	1
	cd	n = 918	199	719	0.217	8.0	0.02	4.0-12.0	1
**Age#**									
≤ 39 years	pd	n = 933	181	752	0.194	4.6	0.019	0.8-8.4	0.8
	cd	n = 956	220	736	0.23	10.0	0.02	6.0-14.0	1
> 39 years	pd	n = 844	193	651	0.229	8.9	0.022	5.6-14.0	1
	cd	n = 885	196	689	0.222	8.7	0.021	4.6-12.8	1
**Competition performed within the last 12 months**									
yes	pd	n = 1,523	328	1,195	0.215	7.8	0.016	4.7-10.9	1
	cd	n = 1,577	348	1,229	0.221	8.6	0.016	5.5-11.6	1
no	pd	n = 280	51	229	0.182	2.8	0.034	-3.9-9.6	0.22
	cd	n = 284	75	209	0.264	15.0	0.039	7.4-22.7	1
**Use of legal/ freely available substances**									
yes	pd	n = 554	119	435	0.215	7.7	0.026	2.6-12.8	0.93
	cd	n = 574	130	444	0.227	9.4	0.026	4.3-14.5	0.98
no	pd	n = 1,239	258	981	0.208	6.7	0.017	3.4-10.1	1
	cd	n = 1,284	290	994	0.226	9.4	0.017	5.9-12.8	1
**Use of analgesics during the last three months**									
yes	pd	n = 198	48	150	0.242	11.8	0.045	2.9-20.7	0.88
	cd	n = 209	34	100	0.254	13.5	0.056	2.5-24.5	0.84
no	pd	n = 1,605	331	1,274	0.206	6.4	0.015	3.5-9.4	1
	cd	n = 1,659	385	1,337	0.224	9.0	0.015	6.1-11.9	1
**Overall hours of training per week#**									
≤ 8 hours	pd	n = 995	216	779	0.217	8.0	0.02	4.2-11.9	1
	cd	n = 1,028	260	768	0.253	13.4	0.02	9.4-17.3	1
> 8 hours	pd	n = 807	163	664	0.197	5.1	0.021	1.0-9.1	0.82
	cd	n = 839	163	676	0.194	4.7	0.02	0.7-8.6	0.78

Factors associated with physical (pd, grey-background) and cognitive doping (cd, white-background) are presented separately, as are the percentage of 'yes' answers from the sub-population. For all 'yes' answers (a), the standard error (SE), confidence interval (CI) and the calculated prevalence estimates ($\hat{\pi}_s$) are provided. The - continuous scaled variables that are marked '#'- were dichotomised by median and again a Post-hoc power analyses (Power) was performed.

© 2019 by the authors. Submitted for possible open access publication under the terms and conditions of the Creative Commons Attribution (CC BY) license (http://creativecommons.org/licenses/by/4.0/).
